# Test evaluation trials present different challenges for trial managers compared to intervention trials

**DOI:** 10.1186/s13063-020-04861-7

**Published:** 2020-11-30

**Authors:** Caroline Rick, Sue Mallett, James Brown, Ryan Ottridge, Andrew Palmer, Victoria Parker, Lee Priest, Jonathan J. Deeks

**Affiliations:** 1 Nottingham Clinical Trials Unit Building 42, University of Nottingham, Nottingham, NG7 2RD UK; 2 UCL Centre for Medical Imaging, University College London, London, UK; 3 Birmingham Clinical Trials Unit, University of Birmingham, Birmingham, UK; 4 NIHR Birmingham Biomedical Research Centre, University Hospitals Birmingham NHS Foundation Trust and University of Birmingham, Birmingham, UK; 5 Test Evaluation Research Group, Institute of Applied Health Research, University of Birmingham, Birmingham, UK

**Keywords:** Diagnostic test accuracy, Test evaluation, Randomised controlled trials, Clinical trial, Recruitment, Sensitivity and specificity

## Abstract

**Introduction:**

Test evaluation trials present different challenges for trial managers compared to intervention trials. There has been very little research on the management of test evaluation trials and how this impacts on trial success, in comparison with intervention trials. Evaluations of medical tests present specific challenges, because they are a pivot point bridging the complexities of pathways prompting testing with treatment decision-making. We systematically explored key differences in the trial design and management of test evaluation trials compared to intervention trials at the different stages of study design and delivery. We identified challenges in test evaluation trials that were more pronounced than in intervention trials, based on experience from 10 test evaluation trials.

**Methods:**

We formed a focus group of 7 trial managers and a statistician who had been involved in the day-to-day management of both test evaluation trials and intervention trials. We used discussion and content analysis to group challenges from 10 trials into a structured thematic format. The trials covered a range of medical conditions, diagnostic tests, clinical pathways and conditions including chronic kidney disease, chronic pelvic pain, colitis, detrusor over-activity, group B streptococcal colonisation, tuberculosis and colorectal, lung, ovarian and thyroid cancers.

**Results:**

We identified 10 common themes underlying challenges that are more pronounced in test evaluation compared to intervention trials. We illustrate these themes with examples from 10 trials, including with 31 specific challenges we experienced. The themes were ethics/governance; accessing patient populations; recruitment; patient preference; test processes, clinical pathways and samples storage; uncertainty of diagnostic results; verifying diagnosis (reference standard); follow-up; adverse effects; and diagnostic impact.

**Conclusion:**

We present 10 common themes, including 31 challenges, in test evaluation trials that will be helpful to others designing and managing future test evaluation trials. Proactive identification of potential challenges at the design and planning stages of test evaluation trials will enable strategies to improve trial design and management that may be different from standard strategies used for intervention trials. Future work could extend this topic to include challenges for other trial stakeholders including participants, clinicians, statisticians and funders.

**Trial registration:**

All trials reviewed in this project were registered and are provided in Table 1.

## Background

Test evaluation trials present different challenges for trial managers compared to intervention trials. Clinical trials are the highest standard of evidence for new treatments and interventions and require a significant investment in time and money. Despite this, many trials fail to recruit to target or complete recruitment /follow-up in line with the protocol [1–3]. This failure delays the implementation of effective treatments and may produce sub-optimal data sets that are too small to reliably answer the question posed.

There has been a great deal of research into clinical trial conduct and completion which has focussed on many aspects of trial design, site selection and monitoring, participant recruitment and retention [4–6]. The UK National Institute Health Research (NIHR) and Medical Research Council (MRC) have invested in methodologies to improve trial design and management, particularly recruitment and retention including the MRC START, ORRCA and Trials Forge initiatives [7–9], and encourage studies within trials to address these issues. However, this work has mostly focussed on intervention trials and may not be as relevant for other trial designs.

In recent years, there has been an increase in the numbers of test evaluation trials, trials that assess how correctly a test detects the presence or absence of a target condition. This article discusses test evaluation trials conducted in a clinical trials unit, distinguishing these from test evaluation studies which may have a less strict protocol-directed design, data collection and analysis methods. Trials can evaluate tests in the context of diagnosis, screening, monitoring, impact on therapeutic management, as prognostic tests or by directly measuring patient outcomes. Diagnostic trials can evaluate the accuracy, time to diagnosis, diagnostic yield, number of tests in a patient pathway or other diagnostic outcomes.

These diagnostic evaluation trials fundamentally differ from intervention trials in the relationship to participants, uncertainty for participants and how these trials affect patient care pathways. Designs of diagnostic trials differ reflecting the different trial objectives. For example, for many test evaluation trials, a randomised design is not the best or most efficient design, exemplified by trials comparing diagnostic tests where participants may receive all the tests being compared as well as a reference standard test. In some trials of new tests where the accuracy is not established in clinical practice, the trial will be designed so the new test results are not revealed during patient care to prevent an unproven test influencing patient treatment.

There is a clear need for specific guidance on the design and management of test evaluation trials, identified by differences in the role of study designs, study biases, systematic review guidelines and reporting guidelines for these trials [10–14]. The current literature has focussed on important aspects of design including phases of design and statistical methods [15–17]. Our paper by contrast aims to provide a handy summary of past experience aimed primarily for trial managers to help planning of the practical aspects of diagnostic evaluation trials.

We identified challenges in test evaluation trials that were more pronounced than in intervention trials, using a focus group of trial managers and a statistician, based on our experience of 10 trials managed at Birmingham Clinical Trials Unit (BCTU) or in collaboration with the Test Evaluation Group at the University of Birmingham. Our paper presents 10 themes, mainly aimed at trial managers, to prompt discussion of practical challenges that can arise in a diagnostic evaluation trial. Early recognition of these issues can greatly aid trial management.

## Methods

We explored the views and opinions of a face-to-face focus group at BCTU consisting of 9 individuals including trial managers who had managed both intervention trials and test evaluation trials and senior statisticians experienced in test evaluation trials. We used content analysis to systematically organise our experience of challenges into a structured format. We circulated a plan, including test evaluation trials proposed for inclusion and preliminary identification of different areas of trial management to discuss. Our focus group met face to face four times in a BCTU meeting room. We included all the diagnostic trials on which members of the focus group were currently directly working on as either trial managers or statisticians. In the first meeting, we reflected on the challenges in test evaluation trials which were different from intervention trials, and in turn, each member reflected on the most challenging issues in their trials. Based on the challenges identified, we started to develop themes to systematically group our experiences which were refined over subsequent meetings. Over the following three meetings of 1-h duration (Feb to April 2017), we focused on 3 or 4 themes per meeting, systematically asking focus group members for each trial in turn to identify challenges for the themes. Discussion in the group and examples from other trials often elicited other examples, as common themes were identified between trials. After each meeting, notes were circulated to the focus group for feedback. Challenges were grouped into a list of 10 areas of trial management, and examples from different trials were selected to illustrate issues in each area by two members of the focus group and circulated to the full focus group for feedback. All members of the focus group were invited to participate in authorship or to be acknowledged. Results were circulated to trial chief investigators for feedback. We used the Consolidated Criteria for Reporting Qualitative Studies Checklist (COREQ) [18] to guide the reporting.

## Results

The group consisted of 7 trial managers and a statistician who had a range of experience in the design, management and oversight of trials based on the number of diagnostic trials, time and timing of their involvement (pre-, during or post-recruitment phases). Most members of the group had experience in both intervention trials and test evaluation trials. At least one group member had participated in the management-related issues for the 10 trials considered (see Table 1) that had been managed at BCTU, or in collaboration with the Test Evaluation Group at the University of Birmingham.

**Table 1:** Test evaluation trials

Trial title	Funder	Registration number
BUS - Accuracy of Bladder Ultrasound (BUS) in the diagnosis of Detrusor Overactivity (DO): a study to evaluate if ultrasound can reduce the need for urodynamics [18].	NIHR HTA	BUS: ISRCTN:46820623. Registered on September 18, 2012
eGFR-C - Accuracy of glomerular filtration rate (GFR) estimation using creatinine and cystatin C and albuminuria for monitoring disease progression in patients with stage 3 chronic kidney disease [19].	NIHR HTA	eGFR-C: ISRCTN: 42955626. Registered on October 08, 2013
ElaTION - The Efficacy and Cost effectiveness of Real Time Ultrasound Elastography in The Investigation Of Thyroid Nodules and the diagnosis of thyroid cancer.	NIHR	ElaTION: ISRCTN:18261857. Registered on January 28, 2015
ENDCaP-C - Enhanced Neoplasia Detection and Cancer Prevention in Chronic Colitis: a multicentre test accuracy study [20].	NIHR EME	ENDCaP-C: ISRCTN:81826545. Registered on May 11, 2015
GBS-2 - Accuracy of a rapid intrapartum test for maternal group B streptococcal colonisation and its potential to reduce antibiotic usage in mothers with risk factors.	NIHR	GBS-2: ISRCTN:74746075. Registered on April 16, 2015
MEDAL - Can magnetic resonance imaging scan replace or triage the use of laparoscopy in establishing a diagnosis amongst women presenting in secondary care with chronic pelvic pain? [21]	NIHR HTA	MEDAL: ISRCTN:13028601. Registered on February 20, 2012
IDEA - An observational cohort study to evaluate the clinical utility of current and second-generation interferon-gamma release-assays in diagnostic evaluation of tuberculosis [16, 17]^a^	NIHR HTA	IDEA - Not registered
METRIC - Diagnostic accuracy for the extent and activity of newly diagnosed and relapsed Crohn s disease: Multicentre prospective comparison of magnetic resonance imaging and small bowel ultrasound [22]^b^.	NIHR HTA	METRIC: ISRCTN:03982913. Registered on November 5, 2013
ROCkeTS – Refining Ovarian Cancer Diagnostic Test Accuracy Scores [23].	NIHR HTA	ROCkeTS: ISRCTN:17160843. Registered on June 3, 2015
STREAMLINE-COLON, STREAMLINE-LUNG. Diagnostic accuracy for metastatic disease in newly diagnosed lung and colorectal cancer: Prospective comparison of whole-body magnetic resonance imaging with standard staging pathways-The Streamline Trials [24, 25]^c^	NIHR HTA	STREAMLINE: ISRCTN:23068310. Registered on August 28, 2015

^a^Managed by Hilary S Whitworth with input from J Deeks

^b^Managed by the University College London Clinical Trials Unit, London; senior statistician SM

^c^Managed by the Cancer Research UK & UCL Cancer Trials Centre, London; senior statistician SM

### Ethics/governance

In some test evaluation trials, the timing of providing a patient information sheet to participants for trial recruitment is sensitive. This is because sometimes when tests are ordered, participants have not had a discussion with clinical staff about the full range of conditions that tests are designed to detect or rule out. This complicates much of the documentation given to participants, from the title of the project to explanations in the patient information sheet (PIS).

In some test evaluation trials, test results are not revealed to participants or clinicians in the patient care pathway. Where test results will not be made available in a time frame to benefit the patient, the potential lack of benefit to trial participants needs to be clear and may discourage participation. When the performance of the test is not sufficiently established to use in clinical practice, it is important results are not revealed to protect participants from potential harm. Where trials are designed to collect patient samples to develop and evaluate new tests, explaining the purpose of the trial can be harder as the trial may not list all tests that will be conducted on patient samples, to enable future proofing if a future new biomarker is discovered.

In some trials, ethical concerns mean that test results may need to be revealed to participants during their treatment pathway.

#### Example 1: Impact of trial title

ROCkeTS (Refining Ovarian Cancer Test Accuracy Scores) recruits women with suspicion of ovarian cancer immediately on contact with secondary care. We wanted to send the patient information sheets (PIS) with the clinic appointment letter, to give participants more time to consider joining the trial. As clinicians had not necessarily used the word “cancer” as a potential reason for the referral, there were concerns about causing alarm, and clinicians in some cases refused to send out PIS ahead of the clinic appointment.

Similar issues were also identified in eGFR-C, ElaTION and STREAMLINE. In STREAMLINE, the PIS clearly stated that the patient may not have cancer in the permissions for the extra trial MRI.

#### Example 2: Uncertainty in project pathway

ROCkeTS aims to externally validate the existing tests and risk scores and develop a new risk score on the trial cohort of participants. As the project allowed for a potential change in the tests to be performed on the serum samples, it was harder to explain to participants the purpose of the test evaluation study than an intervention study comparing two treatments.

#### Example 3: Trial test result availability for patient care

In STREAMLINE, whole-body MRI was being evaluated in comparison with standard pathways to detect the presence of metastases at cancer diagnosis. Results from whole-body MRI were made available for patient care, because MRI is an accepted test for the detection of metastases, and all participants received MRI in addition to standard care.

### Accessing patient population or accessing participants

Accessing the patient population in test accuracy trials poses different issues to intervention trials, as participants need to reflect the appropriate balance of the relevant clinical population to allow accurate evaluation of the test at the relevant point in the patient pathway. Typical issues include clinician bias, screening burden and time constraints, requirement for clinician referral and access to participants at a relevant point in the patient pathway.

#### Example 1: Screening burden

In ENDCaP-C, recruitment relied on screening endoscopy lists, where the information was not in a readily accessible format and only a minority of patients are potentially eligible. Some trials may require screening of every available patient, which can amount to thousands of potential participants. In eGFR-C for example, 30,000 patients were screened or approached to recruit 1200.

#### Example 2: Clinician referral required

In ElaTION, radiologists were not able to arrange the requisite repeat appointments directly, leading to communication issues with non-ElaTION referring consultants to follow the correct trial pathway. In STREAMLINE, whole-body MRI is compared to NICE pathways as a cancer staging test, requiring referral to another hospital at some centres. This raises concerns that older patients and patients who are temporarily ill at the time of cancer diagnosis could be less likely to be tested due to burden and perceived benefit, potentially restricting the range of the included population.

#### Example 3: Access to population

In ROCKeTS, the ultimate goal is to use the test in primary care to improve the accurate triaging of women referred to secondary care. Unfortunately, it is only feasible to recruit in secondary care at the point of referral, because of the low prevalence of ovarian cancer in patients seen by GPs. Recruiting of women at referral to secondary care needs to take into account that hospital referrals include women referred from both primary care and other secondary care disciplines. In addition, the referral population varies between secondary or tertiary care hospitals, complicating the screening of participants.

### Recruitment

To ensure that the relevant clinical population is used to evaluate a test, recruiting a consecutive series or representative (unselected) population of eligible participants is the ideal. Further complexities result because recruitment often requires collaboration between specialist disease clinical staff and general clinical teams not invested in the trial such as radiologists and pathologists. Variations in the diagnostic pathway between hospitals can also require an understanding of local pathways for recruitment.

#### Example 1: Spectrum bias

In IDEA, adults presenting with suspected active tuberculosis (TB) to NHS outpatient or inpatient services were included. However, due to an error in the implementation of recruitment criteria at one site, only patients with confirmed or highly probable TB were recruited. These participants were excluded from the trial analysis [19, 20].

#### Example 2: Complex pathways

In ElaTION, eligible patients were often not approached because they had been referred from primary care where it is the responsibility of the GP to review the radiology report and decide on the next action. Trial staff needed to create their own clinic list in order to recruit patients. In ROCkeTS, women are eligible based on ultrasound scan and/or abnormal CA125 test results in combination with symptoms. Different hospitals organise ultrasound scanning in different ways, some as a one-stop shop for women with symptoms of ovarian cancer, whereas other hospitals have a general ultrasound service for all referred patients regardless of the referral reason. This affects whether consecutive recruitment is feasible.

#### Example 3: Incompatible pathways

In eGFR-C, eligibility was confirmed by a repeated kidney function test within a given time interval which was expected to be met by the advised routine practice. However, as the test was often not repeated in real-world clinical practice, many potentially eligible patients did not meet the criteria.

### Patient preferences

The burden on participants and the invasiveness of testing during diagnostic trials can mean that not all participants complete all scheduled tests. A fuller assessment of patients’ preferences and issues during diagnostic trials involving patients would be important for future research.

#### Example 1: Research burden

In STREAMLINE, efficient recruitment for whole-body MRI requires pre-reserved slots on MRI, so standard NHS services are not affected by research trials. For some participants, the burden of travelling to a different hospital for a specialised MRI may have affected recruitment. In eGFR-C, the burden of a 3-year follow-up required for the reference test discouraged some participants from completing the study.

#### Example 2: Invasiveness of testing

In ENDCaP-C, a second invasive colonoscopy test may be required solely for trial purposes, which led to challenging management and participant loss to follow-up.

### Test processes, clinical pathways and sample storage

In test evaluation trials, pathways are often complex involving multiple teams which can create logistical barriers to recruitment, completing clinical contact and also in patient follow-up. While the group agreed that many of these barriers also occurred in intervention trials, they felt that they were particularly challenging in a test evaluation setting where participants were undergoing additional tests solely for research purposes.

Test evaluation trials routinely produce a combination of data and accompanying test samples, e.g. tissue, blood, images, which are also a valuable resource for future research. Curating and futureproofing these collections are an important part of the design and may lead to data being collected with a view to future use rather than the immediate project. In the immediate project, standardisation can be required in imaging tests for the format of image collection, machine resolution, anonymization and measurements made during imaging. This standardisation may be to stricter standards than will be done in routine practice which may affect how the test performs in the real world.

#### Example 1: Specialised staff training for test

In ROCkeTS, at some sites, only a sub-set of sonographers was trained to perform one of the tests, and adjusting the normal appointment process to ensure that trial participants were referred to specific sonographers was challenging.

#### Example 2: Preparation and handling of tissue samples

In ROCkeTS, samples are tested in batches on a single platform, and given the number of tests, new sets of reagents will be used; this is unlikely to reflect normal practice where samples are run on an ad hoc basis. Similarly, in eGFR-C, all samples are tested at one of two central laboratories rather than in local hospital laboratories.

#### Example 3: Specialised test processes

In STREAMLINE, whole-body MRI was part of one diagnostic pathway identifying cancer metastasis. The trial protocol specified a minimum of image sequences, but the local hospital decided on the MRI platform (manufacturer and Tesla strength) and imaging parameters. To maintain blinding of MRI images and interpretation to the standard diagnostic pathway, software was installed to upload the MRI images to provide a secure data upload to a secure central imaging server. MRI images were downloaded to the local hospital PACS systems after relevant trial reports were completed.

#### Example 4: Logistics

In IDEA, blood samples to be tested using interferon gamma release assays for active TB required laboratory processing within 8 h. Rapid transport was needed to laboratories in London, Leicester and Birmingham and meant that participants could only be recruited from morning clinics only as the laboratory was not open 24 h.

### Uncertainty of test results

Clinicians can feel that they are being tested personally rather than that the trial is measuring current standard practice, making trial participation and completeness of trial data more challenging. This is more frequent when the trial includes tests requiring interpretation by clinicians where test interpretation is more subjective involving skilled clinical interpretation such as in imaging tests. Reference tests based on consensus panel decisions and trials measuring the diagnostic impact of a test can also make clinicians feel their personal skills are being assessed.

In laboratory tests, uncertainty in test results can be caused by changes in measurement methods in trials with a long follow-up. This can affect tests and reference standards and may require careful consideration.

#### Example 1: Current standard practice vs individual clinician performance

Clinicians feeling their personal skills are being assessed can result in incomplete reporting of data. This can occur in imaging trials and where clinicians are asked to give their opinion or a diagnosis based on the reference standard or incomplete data. This has led to incomplete data being returned, an issue identified in MEDAL, ElaTION and ROCkeTS.

### Verifying diagnosis (reference standard)

In test evaluation trials, a reference standard is used to establish a “true” diagnosis, and all other tests are compared using this “true” diagnosis. The accuracy of the reference standard to correctly identify the target condition impacts how the test being evaluated appears to perform. The reference standard may be another test such as histology or a combination of tests or a consensus panel interpretation of tests.

#### Example 1: Consensus panel reference standard

In MEDAL, the reference standard is the decision of a panel of experts who, despite decision rules, may disagree. Differences in specialist training in combination with how information was presented to the consensus panel may have led to differences in panel member decisions.

#### Example 2: Reference standard tests can include different tests in different participants

In METRIC, additional tests were required in some participants where test results disagreed on the presence or location of the disease. In some participants, an additional test was required to resolve disagreements between tests contributing to the reference standard.

#### Example 3: Availability of reference standard

In GBS, an enriched method of bacterial culture is the reference standard. However, not all sites do this test routinely, resulting in concerns about the need to train microbiologists to interpret the results.

### Follow-up

Follow-up can be particularly challenging in test evaluation trials. Participants may be less willing to return for further “unnecessary” appointments or tests that are part of the trial but not part of the normal clinical pathway. Clinicians may be less willing to refer participants for further trial follow-up if this does not reflect the clinical pathway, particularly where participants with negative tests may in normal practice be discharged from clinic follow-up.

Follow-up of participants can be important for different aspects of the trial, depending on the trial objective. For example, in diagnostic accuracy and prognostic studies, follow-up can be part of the reference standard, whereas economic modelling may require follow-up QoL data. In diagnostic impact trials and monitoring trials, follow-up can be necessary to identify how subsequent testing, patient management and outcomes change.

#### Example 1: Change in current practice

In ElaTION, during the trial, clinical guidelines changed to remove a second patient scan that was important for the trial resulting in a drop in referrals. Similarly, the trial GBS may be affected by a change in clinical guidelines. In both instances, these tests formed part of the trial follow-up.

#### Example 2: Referrals

In ElaTION, consultants can decide whether to refer for the repeat test or not which may selectively affect follow-up of participants with a benign disease that contributes to the reference standard.

#### Example 3: Contact with patient

In ENDCaP-C, participants follow different pathways depending on methylation and histology results, so patient follow-up is different for different participants and it is difficult to confirm follow-up visits with participants and trialists until the test results are completed.

### Adverse effects or harms

Adverse effects (AEs) may be less important in test evaluation trials as if the study is investigating tests used in usual care, AEs directly attributable to standard tests are unusual. However, in a complete diagnostic pathway including treatment, it can be confusing to know how far along the pathway AEs are attributable to tests. In addition, in some situations, AEs caused by testing might be considered important to address.

#### Example 1: Definition of AEs

In ElaTION, AEs can occur during surgery, but participants in both RCT arms have surgery, so it was harder for nurses to understand which AEs should be recorded as directly attributable to the tests and which are assigned to trial treatments.

#### Example 2: Minimising irrelevant AEs

In eGFR-C, AEs are time-limited to 24 h from the test to ensure they are attributable to the test (use of a contrast medium) as most of the 1200 participants are elderly and followed up over 3 years.

#### Example 3: Understanding harm caused by the process of testing

In ROCkeTS, there is a questionnaire capturing stress caused by testing for cancer as a possible condition accounting for symptoms and prior test results.

### Diagnostic impact

The impact of the test can be hard to separate from other parts of the clinical pathway or model. Incremental changes in test accuracy may improve a test but not be sufficient to change practice.

#### Example 1: Measuring impact on patient management

STREAMLINE compares patient management decisions made on the basis of different test pathways during clinical multi-disciplinary meetings for cancer patients. Capturing patient management decisions in a diagnostic trial is a newer design that is cheaper and faster than completing an RCT using a test as an intervention, but is likely to include more potential bias from difficulties in blinding and reporting decisions made in real time in the patient pathway.

#### Example 2: Separating test from complex pathway

In MEDAL, the incremental add-on value of individual components from a complex pathway was assessed using scenarios from trial participants assessed offline from the real patient treatment decisions. Asking clinicians to evaluate the impact of using a test in a theoretical scenario may not be realistic and could inflate the impact of the test in decision-making.

#### Example 3: Implementation

In ROCkeTS, the impact of a new test will depend on GP confidence to change the patient pathway and stop referring patients to secondary care. There is no evidence on the thresholds in diagnostic accuracy that a test would have to perform at to change practice.

## Discussion

We identified 10 common themes underlying the challenges that are more pronounced and in some instances unique in test evaluation compared to intervention trials (Table 2). We illustrate these themes with examples from 10 trials, including with 31 specific challenges we experienced. Some themes were expected such as for reference standards, but others such as challenges accessing patient populations were unanticipated. In most cases, the realisation of these challenges earlier in the trial would have greatly aided trial management.

**Table 2:** Differing themes and challenges from diagnostic test accuracy trials

Themes	Challenges	Description
Ethics/governance	Patients approached for recruitment require information that is sensitive to their knowledge of their disease.	Timing is sensitive as information may need prior discussion with the participant about what condition tests are designed to detect or rule out. This requires careful consideration of the trial title and the patient information sheet.
	Explaining the purpose of the trial, where trial design collects patient samples to evaluate and develop new tests.	Explaining the purpose of the trial to patients can be more complicated where the trial does not list all tests based on patient samples.
	Explaining the purpose of trial developing a new test may lead to questions about deficiencies in the current test pathway.	Explaining the purpose of trial evaluating current tests or developing a new test can be complicated, as it may raise questions about the current diagnostic pathway, its accuracy and uncertainties.
	No direct benefit to participants.	Trial may be about developing a new test, so trial participants have on direct benefit.
	Trial test results availability required for patient care where appropriate.	For ethical reasons, test results may need to be revealed for patient care, particularly in trials comparing tests in current clinical use.
Accessing patient populations	Screening burden.	Eligible patients may need to be screened manually from the hospital clinic list where the clinic conducts a test used to diagnose several diseases, but the trial only relates on one type of referral.
	Accessing patients at a clinically relevant time point.	Hospital clinics receive patients through different referral pathways, only some of which may be relevant to the trial. Hospitals arrange clinical care pathways differently.
Recruitment	Patients eligible for the trial should represent a clinical referral population without clinical selection (spectrum bias)	Requirement for an additional clinician referral required by the trial can affect who gets recruited. Clinicians can misunderstand eligibility criteria and select participants by referral plus their clinical suspicion.
	Recruitment requires collaboration between specialist disease and general clinical teams	Patient recruitment may occur in the radiology department for trial diagnosing a specific disease requiring recruitment in clinics without research staff directly involved in the trial.
	Collaboration across clinical settings	Patient pathway in the hospital may depend on the interpretation of tests made by primary care clinicians.
Patient preferences	Research burden.	Participants may find it difficult to attend additional hospital appointments or travelling to a different hospital site when they are feeling ill, or it takes extra time from family care or work.
	Reference test may be too onerous to patients.	For some participants, follow-up may require an additional invasive or unpleasant test, which may be too burdensome.
Test processes, clinical pathways and sample storage	Trial may require a change in clinic patient pathway or referral to specialist test clinic.	Trial pathway may require referral to staff trained in a new test method. Test may not be available in a recruiting centre but may take place at a specialist hospital clinic.
	An extra test or patient sample is required for trial.	Different clinics may need to order or interpret the test, resulting in delays or reduced recruitment.
	Test may require extra staff training to conduct or interpret test results.	Can restrict recruitment to time periods when staff with specialist test training are on duty. Extra staff training may be required.
	Trial includes standardisation of test processes to a higher standard than normal clinical practice.	Tests may require additional burden on clinics of specialist equipment or calibration of equipment or additional software or extra clinical interpretation.
	Patient sample may need immediate testing by a specialist laboratory.	Recruitment sites had to be within a certain distance of laboratory to enable sample testing within a required time.
	Requires additional requirements for collection and storage of trial samples, in addition to normal clinical practice.	Additional burden to clinics including anonymisation of images, cloud/server storage of images, sending samples, collation of patient information for interpretation at a second site, etc.
	Trial may store samples of blood or tissue for future test development.	Requires curation and future proofing tissue storage.
Uncertainty of test results	In tests requiring interpretation by clinicians, clinicians can feel their ability is being tested, rather than the test.	Clinicians may be cautious or report some data intermittently if they feel the trial is measuring their performance rather than the performance of the test in a typical clinical setting.
	Calibration of the index and reference tests across time can be problematic.	Calibration and standardisation of tests and equipment over time can affect diagnostic performance.
Verifying diagnosis (reference standard)	Reference standard may require formation of an expert consensus group to review and interpret patient diagnosis based on several tests.	Requires extra time from staff and clinicians to prepare and attend meetings. Processes to resolve disagreements are needed.
	Reference test may depend on the results from the index tests.	Additional tests may be required when index tests disagree.
	Reference test requires a specialist test.	If a specialist test is required as part of the reference test, it may not be available at all hospital sites and may require specialist training.
Follow-up	Patient follow-up is often needed to confirm disease status.	Participants may be unwilling to return for “unnecessary” appointments. Clinicians may be unwilling to refer participants for additional follow-up tests not part of usual clinical practice. Clinical guidelines can change and affect tests forming part of trial follow-up.
	Follow-up tests and procedures can be different for participants, depending on their test results during the trial.	Follow-up for a patient can vary from further imaging, surgery, histology of sample from surgery to follow-up of primary care records.
	Follow-up may require collaboration across clinics or healthcare settings.	Follow-up in participants with no disease may require follow-up through primary care, patient contact or national databases.
Adverse effects or harms	Can be difficult for clinical staff to understand which AE in the clinical pathway should be recorded as relevant to the trial.	Discussion of AE relevant to the trial needs careful definition differentiating AE from the test itself or AE in patient pathway alternation by use of the test.
	AE can be caused by consenting a patient for testing within a trial.	Informed consent may make participants more aware of the range of diagnoses from tests than discussed in clinical practice. Trial participation may also prompt more discussion about diagnostic uncertainty from current tests.
Diagnostic impact	Measuring the impact of tests on patient management.	Patient management decisions can be captured within diagnostic accuracy trials. The highest level of evidence is from an RCT of using tests as an intervention, but alternative designs reporting patient management decisions instead of patient outcomes can, for some tests, provide important evidence on test impact faster and at a lower cost.
	Understanding the incremental benefit of a particular test within a complex patient pathway is difficult.	Separating a test to measure its individual impact can become artificial.
	Understanding what difference in diagnostic accuracy or diagnostic confidence is required to change clinical practice.	Often adding a test into a test pathway has an incremental value on certainty or timing of diagnosis.

Design and management of test evaluation trials clearly pose different challenges to intervention trials. Recognition of specialist skills needed and training for CTUs in design and management of test evaluation trials will be important to increase success. Having a clear understanding of these specialist challenges allows strategies to address them to be incorporated into the project management plan to improve the trial delivery. These strategies may well start at the grant application stage and influence many aspects of the design and management. As with intervention trials, the effectiveness of the strategies needs to be reviewed on an ongoing basis during the lifecycle of the trial.

This project focus group considered 10 test accuracy trials across a range of diseases managed at BCTU or analysed within the Test Evaluation Group at the University of Birmingham. Including 10 trials and 8 trialists was a limitation of our study; a broader range of trials or opinions from trial managers at other trials units may have identified additional issues. The current literature on trial management focusses on interventional trials and we are not aware of other studies providing this practical trial management experience for diagnostic accuracy trials to aid future trials and trialists. This paper focused on trial management and did not aim to include all issues in test evaluation trials relevant to clinicians, research nurses, patients, statisticians and other stakeholders. There clearly is the need for a larger project on this topic including these other perspectives.

This paper provides important insight into improving the design and delivery of clinical trials in test evaluation, as well as identifying the need for further work.

## Conclusion

Test evaluation trials are fundamental to the improving diagnosis of disease and decision-making for efficient and effective patient treatment pathways. Ensuring the quality of data and the clinical relevance of the recruited participants are dependent on trial design and management practices that are tailored specifically to test evaluation trials.

We provide a list of 10 themes, including 31 challenges, to prompt discussion of practical challenges that can arise in a diagnostic evaluation trial. Early recognition of these issues can greatly aid the design and management of future test evaluation trials.

## Data Availability

Not applicable. The trials discussed in this publication will be published or have been published separately. This project did not generate data or materials.

## Abbreviations

BCTU: Birmingham Clinical Trials Unit
AEs: Adverse effects
MRC: Medical Research Council
NIHR: UK National Institute Health Research
PIS: Patient information sheet
GP: General practitioner (primary care physician)
DTA: Diagnostic test accuracy
